# Deep muscularis propria tumor invasion without lymph node metastasis as a unique subclassification of stage IB gastric cancer: a retrospective study

**DOI:** 10.1186/s12876-021-02090-z

**Published:** 2022-01-21

**Authors:** Kang He, Cheng Chen, Lei Xia, Lixiang Si, Xiaohua Pan, Zijian Sun, Yajing Wang, Yingying Jiang, Yue Shi, Bin Zhou, Shuaiyu Wang, Jing Han, Bo Shen, Guoren Zhou, Jianwei Lu, Xiaohua Wang

**Affiliations:** 1 The Department of Oncology, The Affiliated Cancer Hospital of Nanjing Medical University, Jiangsu Cancer Hospital and Jiangsu Institute of Cancer Research, Nanjing, People’s Republic of China; 2 The Department of Radiotherapy, The Affiliated Cancer Hospital of Nanjing Medical University, Jiangsu Cancer Hospital and Jiangsu Institute of Cancer Research, Nanjing, People’s Republic of China; 3 The Department of Pathology, The Affiliated Cancer Hospital of Nanjing Medical University, Jiangsu Cancer Hospital and Jiangsu Institute of Cancer Research, Nanjing, People’s Republic of China; 4 The Department of General Surgery, The Affiliated Cancer Hospital of Nanjing Medical University, Jiangsu Cancer Hospital and Jiangsu Institute of Cancer Research, Nanjing, People’s Republic of China; 5 UCL School of Pharmacy, University College London, London, UK; 6 The Department of Epidemiology, The Affiliated Cancer Hospital of Nanjing Medical University, Jiangsu Cancer Hospital and Jiangsu Institute of Cancer Research, Nanjing, People’s Republic of China

**Keywords:** Deep muscularis propria, Superficial muscularis propria, Stage IB gastric cancer, p53, Serum tumor marker, Prognosis

## Abstract

**Background:**

The prognosis difference based on the depth of tumor muscularis propria invasion in gastric cancer (GC) was still debated, and therapy strategy for stage IB GC patient required further investigation.

**Methods:**

A total of 380 patients with pT2 GC after radical surgery were retrospectively analyzed, including 185 in superficial muscularis propria (sMP) group and 195 in deep muscularis propria (dMP) group.

**Results:**

The overall survival (OS) was significantly better for patients in sMP group than for patients in dMP group (P = 0.007). In multivariate analysis, depth of tumor invasion, pN stage, age, primary location, positive expression of p53, elevated maximal LDH, elevated initial CA19-9 and AFP level were independent prognostic factors for OS. The sMP group had a significantly better OS than dMP group (P = 0.014) in pN0 stage. After further stratification, the survival outcomes were not significantly different between deep muscularis propria tumor invasion without lymph node metastasis (dMPN0) group (stage IB) and superficial muscularis propria tumor invasion with stage 1–2 lymph node metastasis (sMPN1–2) group (stage II) (P = 0.100). Patients with adjuvant chemotherapy had a statistically better survival than those without in dMPN0 group (P = 0.045) and dMPN0 patients with adjuvant chemotherapy had better OS than sMPN1–2 patients (P = 0.015). In addition, greater postoperative survival could be observed in sMPN0 patients than dMPN0 patients in p53-positive group (P = 0.002), and similar OS could be seen between dMPN0 patients with p53-positive and T2N1–2 patients (P = 0.872).

**Conclusion:**

As a unique subclassification of stage IB GC, appropriate adjuvant chemotherapy should be considered for patients with dMPN0 stage. In addition, positive expression of p53, elevated LDH could be potential factors in identifying the different prognoses for stage IB GC patients.

**Supplementary Information:**

The online version contains supplementary material available at 10.1186/s12876-021-02090-z.

## Introduction

Gastric cancer (GC) is the third commonest malignant disease and the fourth most frequent cause of cancer-related deaths in the world. With the growing progression of the medical technology and the improvement of health consciousness, the incidence of gastric cancer declined steadily, however, the prognoses of GC patients are still pessimistic [1–3].

Accurate staging of gastric cancer is the basis for guiding treatment strategy and judging the prognosis of patients. TNM staging system introduced by the Union for International Cancer Control/American Joint Committee on Cancer (UICC/AJCC) is adopted internationally in recent years. However, in clinical practice, the prognosis for pT2 staging GC, a variant of advanced gastric cancer, is diverse, which could not be simply explained by the TNM staging and postoperative therapy regimens. In general, the muscularis propria of the stomach is histologically subdivided further into two layers: the inner circular layers, and outer longitudinal layers. In addition, the newest edition of TNM stage system does not specify details for definition of subclassification of pT2 stage (sMP vs. dMP). Therefore, we believe that it is reasonable to consider that the subclassification of the pT2 stage can be used as a new standard for prognostic prediction and clinical decision-making.

The prognosis for gastric cancer differs widely even among patients with the same tumor stage and grade. The TNM classification does not completely differentiate good and bad prognosis of individual patients. Therefore, there is an urgent need for new biomarkers, such as serum tumor markers and gene mutations, improving the assessment accuracy of the pTNM staging and tumor aggressiveness. Currently, it is thought that the accumulation of mutated genes results in GC tumorigenesis, and several gene mutations, such as p53, HER-2, EGFR, VEGF, have been proved to be correlative with the prognosis of gastric cancer in several studies. In addition, serum tumor markers, like carcinoembryonic antigen (CEA), lactate dehydrogenase (LDH), carbohydrate associated antigen 19-9 (CA19-9), cancer antigen 125 (CA125) and alpha-fetoprotein (AFP), are more convenient and cost-effective detections than other approaches, they are extensively used in monitoring disease condition, assessing treatment effect, estimating prognosis and predicting recurrence [4].

Above all, we conduct present study to identify the relations between subclassification of pT2 gastric cancer and survival outcomes and clinicopathological features according to the depth of tumor involvement, and further analyze potential markers to reinforce the prognostic and therapy-guided ability of the TNM staging system.

## Materials and methods

### Ethics statement

The study was approved by the clinical research ethics committee of the Jiangsu Cancer Hospital and was conducted in accordance with the Declaration of Helsinki.

### Patients

**Fig. 1:**
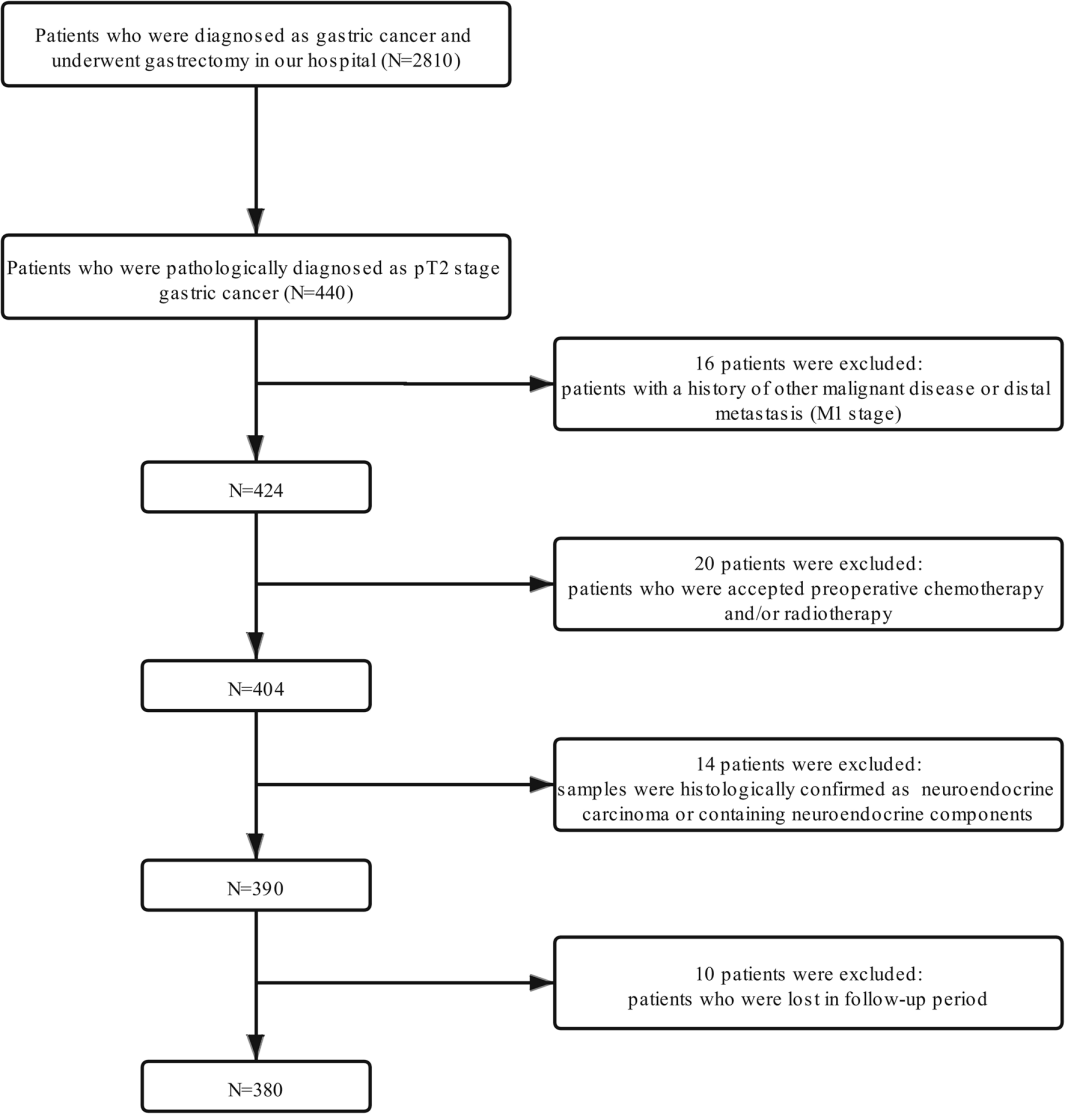
Flow diagram of the patient selection process

A total of 2810 patients underwent curative gastrectomy between January 1, 2008 and December 31, 2014 were retrospectively collected from our institutes. The inclusion criteria were: (1) a tumor pathologically diagnosed as gastric adenocarcinoma; (2) patients who accepted radical gastrectomy (R0) and pathologically were confirmed as pT2N0–3M0 stages according to the 8th edition TNM staging system (UICC/AJCC); (3) clinicopathological data and follow-up information were complete. The exclusion criteria were: (1) patients with a history of distant metastases and/or other malignant diseases; (2) patients who accepted preoperative chemotherapy and/or radiotherapy; (3) tumors that were pathologically confirmed as neuroendocrine tumors or contained neuroendocrine components. Detailed flow chart for selection process could be seen in Fig. 1. Finally, 380 patients who met the inclusion but the exclusion criteria were analyzed in our study. Patients were separated into the sMP group and the dMP group based on the tumor involvement depth by two pathologists. In line with previous study [5], the sMP was defined as the maximal invasive depth of tumor to the superficial half part of the MP layer (inner circular muscle bands), while the dMP was defined as the maximal invasive depth of tumor to the deep half part of the MP layer (outer longitudinal muscle bands) (Additional file 1: Fig. S1).

### Therapy strategy

According to the treatment guidelines and preoperative examination (radiological imaging tests and pathological tests), patients who matched surgical indications in our hospital underwent gastrectomy with standard lymph node dissection. The decision as to whether patients received postoperative adjuvant chemotherapy depended on the integrated evaluation of pathological examination, therapy guideline and patient intention. Adjuvant chemotherapy regimens were single-agent fluoropyrimidine or platinum combined with fluoropyrimidine.

### Immunohistochemistry

Immunohistochemistry (IHC) were performed as previously described [6, 7], detailed experimental steps were as follows. Four-micrometer-thick sections were obtained from formalin-fixed, paraffin-embedded tumor tissues. Tumor sections were deparaffinized in xylene and rehydrated through graded alcohols. Gastric slides were immersed and heated in a 0.01 M sodium citrate buffer (pH 6.0) at 121 °C for 2 min to repair antigens. Endogenous peroxidase activity was blocked through immersing the slides in 3% hydrogen peroxide for 10 min. The slides were then washed 3 times for 3 min each time with phosphate buffered saline (PBS). Tissue samples were finally exposed in non-immune horse serum for 30 min to reduce non-specific binding. The primary antibody was monoclonal mouse anti-human antibody (Do-7, Dako, Denmark, 1:100) for p53, monoclonal rabbit anti-human antibody (MXR001, MXB-BIO, China, 1:100) for HER-2, monoclonal rabbit anti-human antibody (EP22, MXB-BIO, China, 1:100) for EGFR, monoclonal mouse anti-human antibody (VG1, MXB-BIO, China, 1:100) for VEGF. Sections were incubated with the primary antibody overnight at 4℃, then incubated with a secondary antibody for 30 min at room temperature. After further washes in PBS, a diaminobenzidine tetrahydrochloride (DAB) solution was applied for visualization. Finally, the sections were counterstained with hematoxylin. Sample of positive-stained gastric cancer was served as positive control and PBS was used as a negative control. HER-2 scores were calculated according to Hofmann et al.’s criteria [8], HER2 positivity in the present study was defined as an IHC score of 2+ and 3+. In addition, EGFR and VEGF staining were interpreted as positive when > 10% of the tumor cells stained and p53 when > 30% showed distinct nuclear staining. Representative IHC pictures of each biomarker were shown in Additional file 1: Fig. S2.

### Study parameters

Clinicopathologic features include gender (male, female), age (≤ 60, > 60), smoking history (yes, no), drinking history (yes, no), ECOG score (0–2), location of primary tumor (upper, middle and lower), Borrmann type (Type I–II, III–IV), pathological type (adenocarcinoma, mucinous/rare carcinoma), histologic type (well and moderate, poor), neural invasion (negative, positive), lymphovascular invasion (negative, positive), pN stage (N0-N3), TNM stage (I–IIIA), adjuvant chemotherapy (without, with). Molecular markers including HER-2/neu, p53, VEGF, EGFR (negative, positive). Initial tumor markers were detected within seven days before surgery and maximal LDH was defined as the maximum of LDH during follow-up. The cut-off value of LDH, CEA, CA125, CA19-9 and AFP levels were 245 U/L, 3.5 ng/ml, 35 U/ml, 39 U/ml and 7 ng/ml. Pathological tumor staging was based on the TNM staging system of UICC/AJCC (eighth edition).

### Statistics

Continuous and categorical variables were assessed through the t-test, Chi-squared test or Fischer’s exact test, separately. Kaplan–Meier method and log-rank test were performed to distinguish univariate survival outcomes. Variables with P value < 0.1 in the univariate survival analysis were included in multivariate Cox’s proportional hazard model to identify independent prognostic factors. Statistical differences were considered as significant at two-sided P values < 0.05. All statistical analyses were estimated through SPSS software (version 22, Chicago, IL, USA).

## Results

### Patients’ characteristics

Finally, 185 patients in the sMP group and 195 patients in the dMP group were retrospectively analyzed. For clinicopathological factors, neural invasion, and elevated initial AFP level were more likely to appear in patients in the dMP group compared with the sMP group (P = 0.010, P = 0.005, respectively). There were lower number of lymph node metastases in the sMP group than dMP group (1.0 ± 2.1 vs. 1.6 ± 2.7, P = 0.027). There were no obvious differences in sex (P = 0.557), age (P = 0.539), smoking history (P = 0.273), drinking history (P = 0.685), ECOG score (P = 0.334), primary tumor location (P = 0.896), Borrmann type (P = 0.888), pathological type (P = 0.589), histology type (P = 0.836), lymphovascular invasion (P = 0.271), pN stage (P = 0.205), pTNM stage (P = 0.105), number of retrieved lymph node (14.7 ± 6.8 vs. 14.5 ± 6.7, P = 0.889) and postoperative adjuvant chemotherapy (P = 0.138) between the sMP and dMP groups. For molecular markers, the expression of HER-2/neu, p53, VEGF and EGFR were similar in two groups (P = 0.252, P = 0.236, P = 0.315, P = 0.262, respectively). There was no statistical significance in serum tumor markers such as initial and maximal LDH level, initial CEA, CA125 and CA19-9 level between the two groups (P = 0.693, P = 0.165, P = 0.263, P = 0.132, P = 0.126, respectively). Detailed information was shown in Table 1.

**Table 1:** Association between the subclassification of pT2 stage and clinicopathological characteristics of patients

	sMP	dMP	P value
Sex			
Male	146/185 (78.9%)	149/195 (76.4%)	0.557
Female	39/185 (21.1%)	46/195 (23.6%)	
Age			
≤ 60	73/185 (39.5%)	83/195 (42.6%)	0.539
> 60	112/185 (60.5%)	112/195 (57.4%)	
Smoking history			
No	109/185 (58.9%)	104/195 (53.3%)	0.273
Yes	76/185 (41.1%)	91/195 (46.7%)	
Drinking history			
No	111/185 (60.0%)	113/195 (57.9%)	0.685
Yes	74/185 (40.0%)	82/195 (42.1%)	
ECOG score			
0	129/185 (69.7%)	122/195 (62.6%)	0.334
1	42/185 (22.7%)	54/195 (27.7%)	
2	14/185 (7.6%)	19/195 (9.7%)	
Primary location			
Upper	83/185 (44.9%)	92/195 (47.2%)	0.896
Middle	24/185 (13.0%)	25/195 (12.8%)	
Low	78/185 (42.2%)	78/195 (40.0%)	
Borrmann type			
Type I–II	150/185 (81.1%)	157/195 (80.5%)	0.888
Type III–IV	35/185 (18.9%)	38/195 (19.5%)	
Pathological type			
Adenocarcinoma	134/185 (72.4%)	146/195 (74.9%)	0.589
Mucinous/rare carcinoma	51/185 (27.6%)	49/195 (25.1%)	
Histologic type			
Well and moderate	53/185 (28.6%)	54/195 (27.7%)	0.836
Poor	132/185 (71.4%)	141/195 (72.3%)	
Neural invasion			
Negative	174/185 (94.1%)	168/195 (86.2%)	0.01
Positive	11/185 (5.9%)	27/195 (13.8%)	
Lymphovascular invasion			
Negative	162/185 (87.6%)	163/195 (83.6%)	0.271
Positive	23/185 (12.4%)	32/195 (16.4%)	
pN stage			
N0	121/185 (65.4%)	110/195 (56.4%)	0.205
N1	38/185 (20.5%)	45/195 (23.1%)	
N2	19/185 (10.3%)	25/195 (12.8%)	
N3	7/185 (3.8%)	15/195 (7.7%)	
Number of lymph node metastasis*	1.0 ± 2.1	1.6 ± 2.7	0.027
TNM stage			
I	121/185 (65.4%)	110/195 (56.4%)	0.105
II	57/185 (30.8%)	70/195 (35.9%)	
IIIA	7/185 (3.8%)	15/195 (7.7%)	
Number of lymph node retrieved*	14.7 ± 6.8	14.5 ± 6.7	0.889
Adjuvant chemotherapy			
Without	107/185 (57.8%)	98/195 (50.3%)	0.138
With	78/185 (42.2%)	97/195 (49.7%)	
HER-2/neu expression			
Negative	152/185 (82.2%)	151/195 (77.4%)	0.252
Positive	33/185 (17.8%)	44/195 (22.6%)	
P53 expression			
Negative	89/185 (48.1%)	82/195 (42.1%)	0.236
Positive	96/185 (51.9%)	113/195 (57.9%)	
VEGF expression			
Negative	55/185 (29.7%)	49/195 (25.1%)	0.315
Positive	130/185 (70.3%)	146/195 (74.9%)	
EGFR expression			
Negative	65/185 (35.1%)	58/195 (29.7%)	0.262
Positive	120/185 (64.9%)	137/195 (70.3%)	
Initial LDH level (U/L)			
< 245	179/185 (96.8%)	190/195 (97.4%)	0.693
≥ 245	6/185 (3.2%)	5/195 (2.6%)	
Maximal LDH level (U/L)			
< 245	96/185 (51.9%)	115/195 (59.0%)	0.165
≥ 245	89/185 (48.1%)	80/195 (41.0%)	
Initial CEA level (ng/mL)			
< 3.5	143/185 (77.3%)	141/195 (72.3%)	0.263
≥ 3.5	42/185 (22.7%)	54/195 (27.7%)	
Initial CA125 level (U/mL)			
< 35	179/185 (96.8%)	193/195 (99.0%)	0.132#
≥ 35	6/185 (3.2%)	2/195 (1.0%)	
Initial CA19-9 level (U/mL)			
< 39	179/185 (96.8%)	182/195 (93.3%)	0.126
≥ 39	6/185 (3.2%)	13/195 (6.7%)	
Initial AFP level (ng/mL)			
< 7	181/185 (97.8%)	178/195 (91.3%)	0.005
≥ 7	4/185 (2.2%)	17/195 (8.7%)	

*Two-tailed t tests of mean SD. #Two-sided Fisher's exact test, others are two sided χ^2^ test

### Survival analysis

**Fig. 2:**
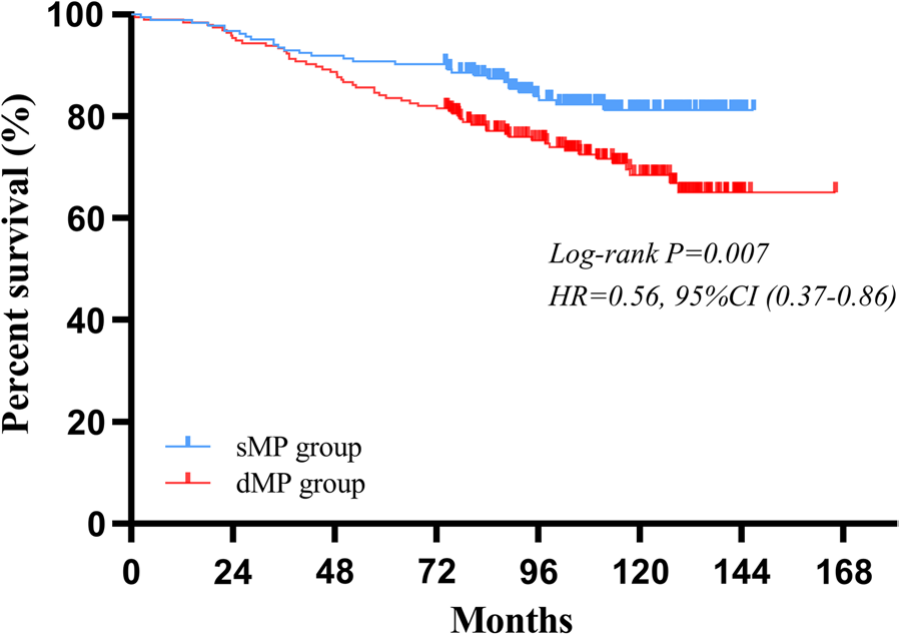
Comparison of the survival outcomes for patients with sMP and dMP gastric cancers

By December 31, 2020, 10 of the 390 cases were lost to follow up during the follow-up. The follow-up period ranged from 2 to 166 months with a median follow-up duration of 99 months. For survival outcomes, patients in sMP group had a statistically better 5-year OS rate than those in dMP group (91% vs. 84%, P = 0.007) (Fig. 2).

Univariate and multivariate survival analyses were used to find prognostic factors (Table 2). Depth of tumor invasion, pN category, age, primary tumor site, neural invasion, positive expression of p53, elevated maximal LDH level, elevated initial CA19-9, CEA and AFP level were statistical prognostic factors in the univariate analysis. Multivariate Cox regression analysis confirmed that depth of tumor invasion, pN category, age, primary tumor site, positive expression of p53, elevated maximal LDH level, elevated initial CA19-9 and AFP level were independent prognostic factors.

**Table 2:** Univariate and multivariate analyses of overall survival according to clinicopathologic factors

	Univariate analysis		Multivariate analysis	
	HR (95% CI)	P value	HR (95% CI)	P value
Sex				
Male versus female	1.014 (0.616–1.669)	0.957		
Age, y				
< 60 versus ≥ 60	2.047 (1.280–3.274)	0.003	2.075 (1.261–3.414)	0.004
Smoking history				
No versus yes	1.119 (0.737–1.700)	0.598		
Drinking history				
No versus yes	0.863 (0.562–1.325)	0.500		
Primary location				
Upper	1.000	0.005	1.000	0.002
Middle	1.020 (0.565–1.844)		0.985 (0.509–1.909)	
Low	0.456 (0.279–0.746)		0.400 (0.235–0.680)	
Borrmann type				
Type I–II versus III–IV	1.482 (0.898–2.445)	0.124		
Pathological type				
Adenocarcinoma versus mucinous/rare carcinoma	0.959 (0.596–1.544)	0.864		
Histologic type				
Well/moderate versus poor	1.163 (0.723–1.871)	0.534		
Neural invasion				
Negative versus positive	1.912 (1.080–3.388)	0.026	1.695 (0.915–3.141)	0.093
Lymphovascular invasion				
Negative versus positive	1.526 (0.899–2.592)	0.117		
Depth of tumor invasion				
sMP versus dMP	1.805 (1.166–2.796)	0.008	1.584 (1.000–2.509)	0.050
pN stage				
N0	1.000	0.000	1.000	0.000
N1	2.543 (1.542–4.193)		2.304 (1.364–3.890)	
N2	2.558 (1.393–4.698)		1.879 (0.967–3.652)	
N3	4.948 (2.502–9.787)		5.335 (2.533–11.237)	
Adjuvant chemotherapy				
Without versus with	0.890 (0.583–1.360)	0.591		
HER-2/neu expression				
Negative versus positive	0.996 (0.593–1.672)	0.987		
P53 expression				
Negative versus positive	2.288 (1.440–3.637)	0.000	1.793 (1.117–2.879)	0.016
VEGF expression				
Negative versus positive	1.069 (0.647–1.768)	0.794		
EGFR expression				
Negative versus positive	0.919 (0.586–1.441)	0.712		
Initial LDH level (U/L)				
< 245 versus ≥ 245	1.230 (0.389–3.890)	0.725		
Maximal LDH level (U/L)				
< 245 versus ≥ 245	1.443 (0.950–2.193)	0.086	1.688 (1.066–2.672)	0.025
Initial CEA level (ng/mL)				
< 3.5 versus ≥ 3.5	1.664 (1.071–2.587)	0.024	0.987 (0.617–1.577)	0.955
Initial CA125 level (U/mL)				
< 35 versus ≥ 35	1.649 (0.521–5.218)	0.394		
Initial CA19-9 level (U/mL)				
< 39 versus ≥ 39	2.450 (1.229–4.883)	0.011	3.572 (1.735–7.358)	0.001
Initial AFP level (ng/mL)				
< 7 versus ≥ 7	2.387 (1.235–4.614)	0.010	2.033 (1.004–4.116)	0.049

*CI* confidence interval, *HR* hazard ratio

**Fig. 3:**
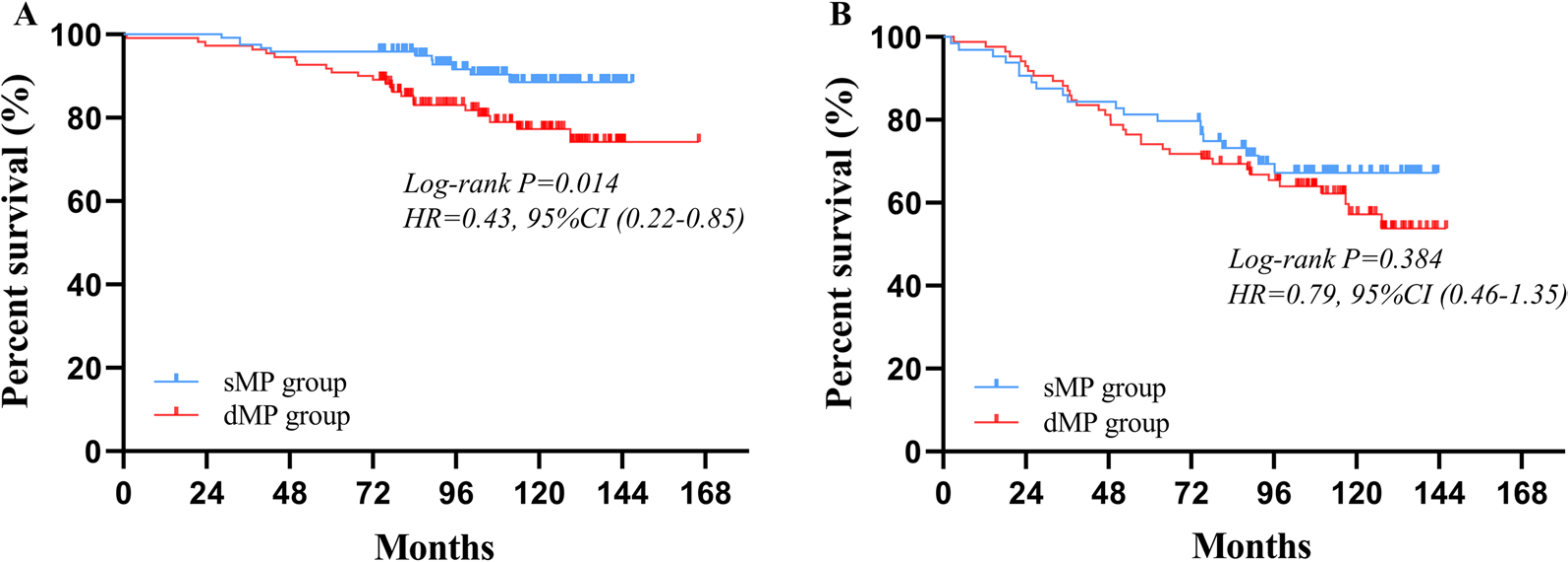
Survival curves of pT2 cancers according to the pN0 staging and pN+ staging, respectively. **A** For patients with pN0 stage tumor, prognosis of the sMP group were significantly different with that of the dMP group (P = 0.014). **B** For patients with pN+ stage tumor, prognosis of the sMP group were not significantly different with that of the dMP group (P = 0.384)

**Fig. 4:**
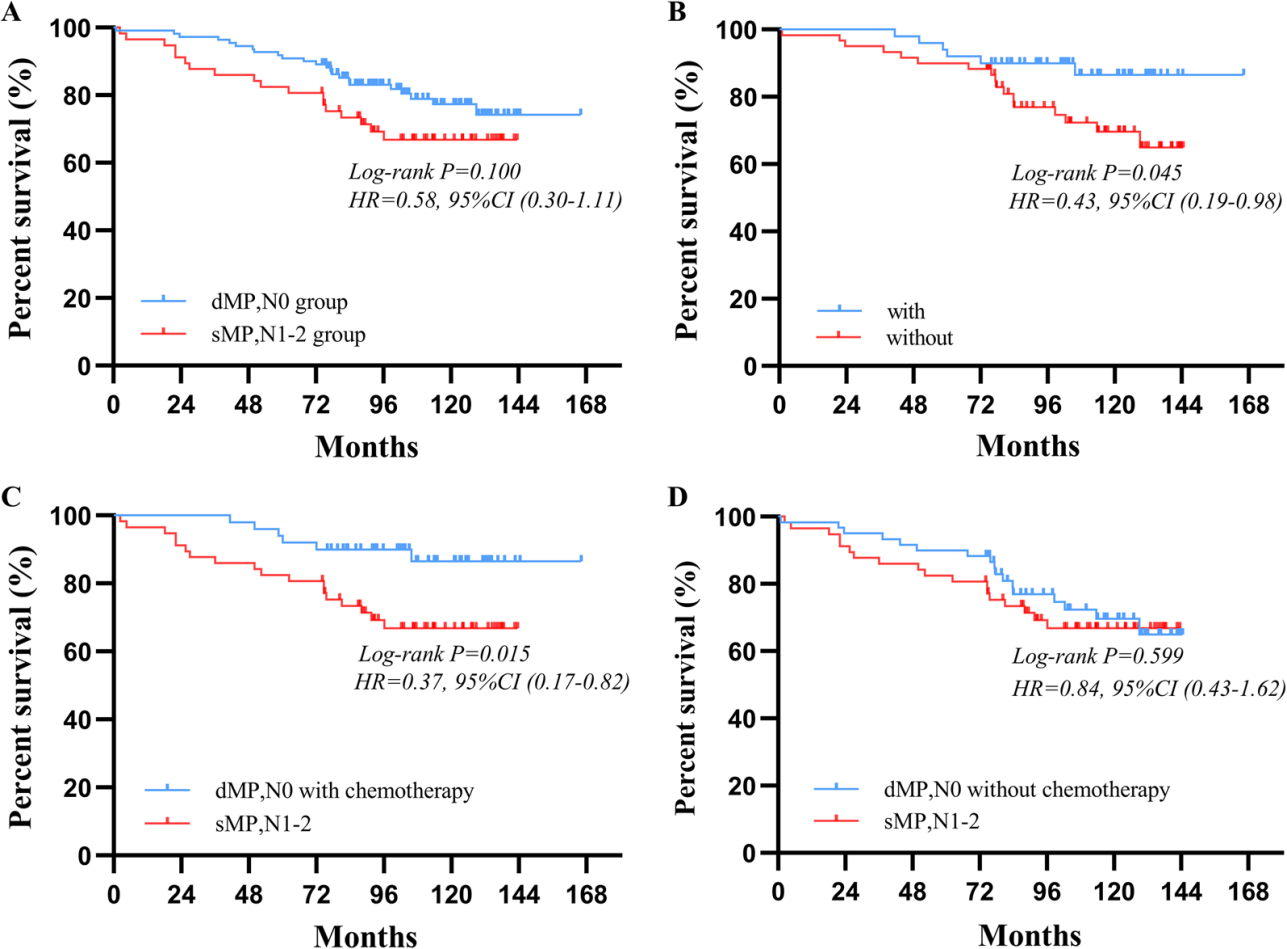
Survival curves of gastric cancer patients in dMP,N0 group and sMP,N1-2 group according to the adjuvant chemotherapy status, respectively. **A** The survival outcome of patients were not significantly different between dMP,N0 group and sMP,N1-2 group (P = 0.100). **B** The survival outcome of patients with and without adjuvant chemotherapy were significant difference in dMP,N0 group (P = 0.045). In comparison to patients in sMP,N1–2 group, patients in dMP,N0 group who accepted adjuvant chemotherapy had better postoperative survival (P = 0.015) (**C**), but not significance in that without adjuvant chemotherapy (P = 0.599) (**D**)

For patients stratified as pN0 stage, significantly better postoperative survival could be observed in sMP group than dMP group (96% vs. 92%, P = 0.014) (Fig. 3A). For patients classified as pN+, there was no obvious differences in the postoperative survival between two groups (80% vs. 74%, P = 0.384) (Fig. 3B). When patients were stratified based on the tumor invasion depth and pN stage, the 5-year OS was no significant difference between the deep muscularis propria tumor invasion without lymph node metastasis (dMPN0) group and superficial muscularis propria tumor invasion with stage 1–2 lymph node metastasis (sMPN1–2) group (92% vs. 82%, P = 0.100) (Fig. 4A). The 5-year OS of patients with the adjuvant chemotherapy were statistically better than those without the adjuvant chemotherapy in the dMPN0 group (94% vs. 90%, P = 0.045) (Fig. 4B), but not significantly better in the sMPN1–2 group (96% vs. 95%, P = 0.204) (Additional file 1: Fig. S3). After further subgroup according to the adjuvant chemotherapy status, in comparison to the sMPN1–2 patients, the dMPN0 patients with the adjuvant chemotherapy had better postoperative survival (82% vs. 94%, P = 0.015) (Fig. 4C), but not significantly better in patients without the adjuvant chemotherapy (82% vs. 90%, P = 0.599) (Fig. 4D).

**Fig. 5:**
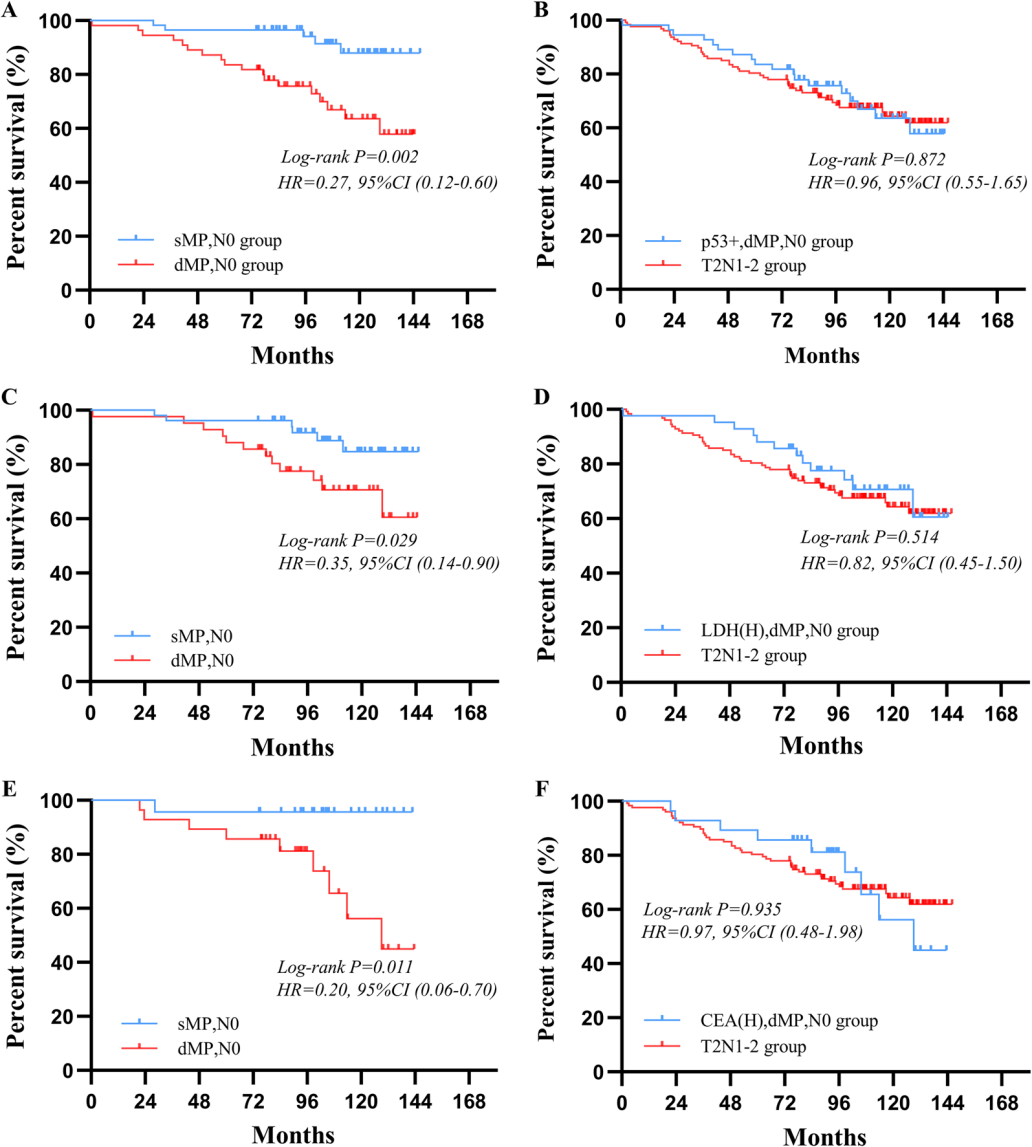
Survival curves of gastric cancer patients in dMP,N0 group and T2,N1–2 group according to other potential prognosis factors, respectively. **A** The survival outcome of sMP,N0 patients were better than dMP,N0 patients in p53-positive group (P = 0.002). **B** The survival outcome of patients in p53+,dMP,N0 group were not significantly different to patients in T2,N1–2 group (P = 0.872). **C** The survival outcome of sMP,N0 patients were greater than dMP,N0 patients in elevated maximal LDH level group (P = 0.029). **D** The survival outcome of patients in elevated maximal LDH,dMP,N0 group were not significantly different to patients in T2,N1–2 group (P = 0.514). **E** There were statistically better overall survival of sMP,N0 patients than dMP,N0 patients in elevated initial CEA level group (P = 0.011). **F** The overall survival of patients in elevated initial CEA,dMP,N0 group were similar to patients in T2,N1–2 group (P = 0.935)

Furthermore, upon stratification of groups according to the expression of p53, in the p53-positive group, greater survival outcomes could be observed in patients with sMPN0 than patients with dMPN0 (97% vs. 84%, P = 0.002) (Fig. 5A). After further comparison, similar survival outcomes could be seen between the dMPN0 patients with p53-positive and the muscularis propria tumor invasion with stage 1–2 lymph node metastasis (T2N1–2) patients (84% vs. 80%, P = 0.872) (Fig. 5B), and no significant difference of 5-year OS was found between p53+, dMPN0 patients receiving and those not receiving adjuvant chemotherapy (91% vs. 87%, P = 0.318) (Additional file 1: Fig. S4). After grouping according to the level of maximal LDH, in the elevated maximal LDH level group, sMPN0 patients had a higher 5-year OS than dMPN0 patients (96% vs. 89%, P = 0.029) (Fig. 5C). There was no significant difference of the 5-year OS between the dMPN0 patients with elevated maximal LDH level and T2N1–2 patients (89% vs. 80%, P = 0.514) (Fig. 5D). Similarly, in elevated initial CEA level group, sMPN0 patients had a better 5-year OS than dMPN0 patients (96% vs. 86%, P = 0.011) (Fig. 5E). Insignificant difference of the 5-year OS could be seen between the dMPN0 patients with elevated initial CEA level and T2N1–2 patients (87% vs. 80%, P = 0.935) (Fig. 5F). The survival outcomes of patients with sMPN0 and dMPN0 were not significantly different in the p53 negative-expression group, normal LDH group and CEA groups.

## Discussion

Despite many indicators were established to calculate the aggressiveness and severity of gastric cancer, pathological TNM (pTNM) stage was still widely used as a critical standard to predict prognosis and guide therapy regimens for GC patients who received curative gastrectomy. However, the survival outcomes for patients with the advanced gastric cancer varied widely even in the same disease stage, similar situation was also seen in pT2 stage. For one thing, the data about clinicopathological characteristics and survival outcomes for pT2 stage gastric cancer patients after radical surgery was limited. For another, it was obvious that cancer aggressiveness, distant metastasis risk and patient prognosis were nearly correlated to the tumor infiltration in gastric cancer [9], and the latest 8th TNM staging system did not define the detailed subclassification of the pT2 stage. Thus, it was necessary to investigate prognostic differences based on the depth tumor muscularis propria infiltration.

For all we know, this was the first study to evaluate differences in the clinicopathologic features and the prognoses of pT2 subclassification based on the depth of tumor muscularis propria infiltration in a large cohort of Chinese gastric cancer patients. Molecular markers and serum tumor markers also were firstly investigated in the pT2 subclassification.

Several studies that discussed the clinicopathological features and survival difference for pT2 stage GC used the older stage system, that contained tumors subserosa invasion stage [5, 10, 11]. On the contrary, our present study was based on the latest staging system that only included tumors invading the muscularis propria, patients were allocated into two groups, the sMP group and dMP group. For general characteristics, there were more regional lymph node metastases in patients of the dMP group than patients of the sMP group, which in line with the previous study [5, 12]. We also found that neural invasion and elevated initial AFP levels were more likely to appear in the dMP group than the sMP group.

In previous studies, Sun and colleagues [5] demonstrated that patients in the sMP group had significantly better survival outcomes than patients in the dMP/SS group, whereas similar outcomes could be observed in the dMP group and the SS group. However, they did not analyze the prognosis differences among the sMP group and dMP group. We demonstrated that in N0 group, patients with sMP tumor had statistically longer survival than patients with dMP tumor, but not in N+ group. This result is consistent with the findings of Zhang and colleagues [12]. In addition, when patients were grouped depending on the depth of tumor invasion and pN stage, we further found that the survival outcomes were not significantly different between patients with the dMPN0 stage and with the sMPN1–2 stage. After further comparison according to postoperative adjuvant chemotherapy status, we observed that significantly improved survival outcomes for patients who had received the adjuvant chemotherapy in the dMPN0 staging group, rather than in the sMPN1–2 staging group. We also observed that the dMPN0 patients with the adjuvant chemotherapy had a better postoperative survival compared to sMPN1–2 patients.

Depending on the latest 8th UICC/AJCC TNM staging system, sMPN0, dMPN0 patients and sMPN1–2 patients were classified as stage IB, IB and II, respectively, and patients with stage II, rather than with stage IB, should receive the adjuvant chemotherapy depending on therapy guideline [13, 14]. In the present study, however, significantly different survival could be seen between sMPN0 group (stage IB) and dMPN0 group (stage IB), but not dMPN0 group (stage IB) and sMPN1–2 group (stage II), and dMPN0 patients who received the adjuvant chemotherapy had obviously improved postoperative survival compared to sMPN1–2 patients. These results showed that the dMPN0 stage should be divided from stage IB as a special subclassification and the dMPN0 patients should receive appropriate adjuvant chemotherapy and follow-up strategy.

As the increase in the understanding of the molecular mechanism of tumorigenesis, it is currently believed that the molecular markers related with the tumorigenesis and disease progression may be potential prognostic factors. The mutated p53 gene subsequently causing inactivation of the p53 protein tumor-suppressor activity appear to constitute one of the commonest molecular steps in tumor development [15, 16]. It is reported that the gene mutation of p53 lead to an increased stability and prolonged half-life time of p53 protein, and result in a nuclear accumulation of protein where it is easily detectable by immunohistochemistry (IHC) using monoclonal antibodies [17]. Although the p53 protein accumulation detected by IHC does not entirely indicate the mutation of gene, the high accordance (85%) could be seen in the p53 overexpression and an underlying mutation [18]. Therefore, the p53 protein overexpression detected through IHC may be considered as a cheaper substitution of that gene mutation. Multiple studies also show the overexpression of the p53 protein as an indicator of poor prognosis in GC [6, 19, 20]. A previous meta-analysis [19], which collected 34 articles focusing on the prognostic value of p53 protein, suggested that positive/high p53 protein expression as a powerful biomarker to predict poorer survival outcomes for gastric cancer patients. Surprisingly, we found that almost all previous studies [6, 19, 20] that discussed the prognostic value of p53 had not performed subgroup analysis based on a certain TNM staging. In our study, we demonstrated that positive expression of p53 protein was a negative prognostic factor for the OS in the early stages of GC. Specifically, for patients with p53-positive, the sMPN0 group had better survival outcomes than dMPN0 group. Moreover, similar survival outcomes could be seen between the dMPN0 patients (stage IB) with p53-positive and T2N1–2 patients (stage II). Although we not found significant survival difference between dMPN0 patients with p53+ receiving and those not receiving adjuvant chemotherapy, but due to the small sample size (55 patients) of the subgroup analysis, the result remained open to question and needed to be validated by a larger sample size research. In summary, the above results might unveil that dMPN0 patients (stage IB) with positive expression of p53 might potentially benefit from adjuvant chemotherapy like patients in stage II.

HER-2/neu as a predictive factor for the therapeutic effect of trastuzumab in the treatment of gastric cancer already has been confirmed by ToGA study [21]. However, the prognostic value of HER-2/neu for GC was still debated. Several studies reported overexpression of HER-2/neu protein as a predictor for aggressive tumor behavior and poor prognosis [22, 23], while others were not [24, 25]. In our study, overexpression of HER-2/neu protein was not an adverse prognostic predictor.

Prognostic value of serologic tumor markers was also been investigated in pT2 GC patients. We demonstrated that the elevated maximal LDH level, elevated initial CA19-9 and AFP level were poor prognostic factors in the multivariable survival analysis, which was concordant with previous studies [4, 26, 27]. Petrelli et al. [28] used a meta-analysis and confirmed that a high serum LDH concentration (> 245 U/L) was associated with a poorer survival in the GC patients. Fanotto et al. [29] found similar results. After further subgroup analysis, we noticed that patients with the sMPN0 had a higher overall survival than those with the dMPN0 in the elevated maximal LDH level group and there was similar OS between the dMPN0 patients with the elevated maximal LDH level and T2N1–2 patients. Similar results also were shown in the dMPN0 patients with elevated maximal CEA levels. According to the above conclusions, we confirmed that serologic tumor markers could be used to identify individual heterogeneity and improve the survival prediction ability of the TNM staging.

Despite some promising findings, our present study still has several limitations. On the one hand, although the sample size of this study is the largest amongst other studies focusing on the T2 subclassification to date, as a single center retrospective study, the results are susceptible to selection bias. Further multi-center, large-sample, prospective studies are therefore required to verify the findings of this study. On the other hand, IHC has been applied to detect molecular markers of cancer in our present study. However, the differences of the types of antibody, concentration and evaluation standard of positivity used in IHC might produce potential bias.

## Conclusion

As a unique subclassification of stage IB GC, appropriate adjuvant chemotherapy should be considered for patients with dMPN0 stage. In addition, positive expression of p53, elevated LDH could be potential factors in identifying the different prognoses for stage IB GC patients.

## Supplementary Information

**Additional file 1. Fig. S1**: Distinction of sMP (inner circular muscle bandsand) and dMP (outer longitudinal muscle bands) invasion according to the maximal invasive depth of tumor. A. tumors limited to sMP layer. B. tumors invaded to dMP layer. CA, cancer.

**Additional file 2. Fig. S2**: Representative case of IHC expression. Cases of IHC expression with (A) Her-2-, (B) Her-2+, (C) p53-, (D) p53+, (E) VEGF-, (F) VEGF+, (G) EGFR-, (H) EGFR+.

**Additional file 3. Fig. S3**: Comparison of the prognoses for sMPN1-2 patients according to the status of adjuvant chemotherapy.

**Additional file 4. Fig. S4**: Comparison of the prognoses for dMPN0 patients with positive expression of p53 according to the status of adjuvant chemotherapy.

## Data Availability

All data generated or analyzed during this study are included in this published article. The datasets used and/or analyzed during the current study are available from the corresponding author on reasonable request.
