# Supplementary figures and images for: Deep muscularis propria tumor invasion without lymph node metastasis as a unique subclassification of stage IB gastric cancer: a retrospective study
Source: BMC Gastroenterol. 2022 Jan 21;22:30. doi: 10.1186/s12876-021-02090-z (PMC8783482; doi:10.1186/s12876-021-02090-z)

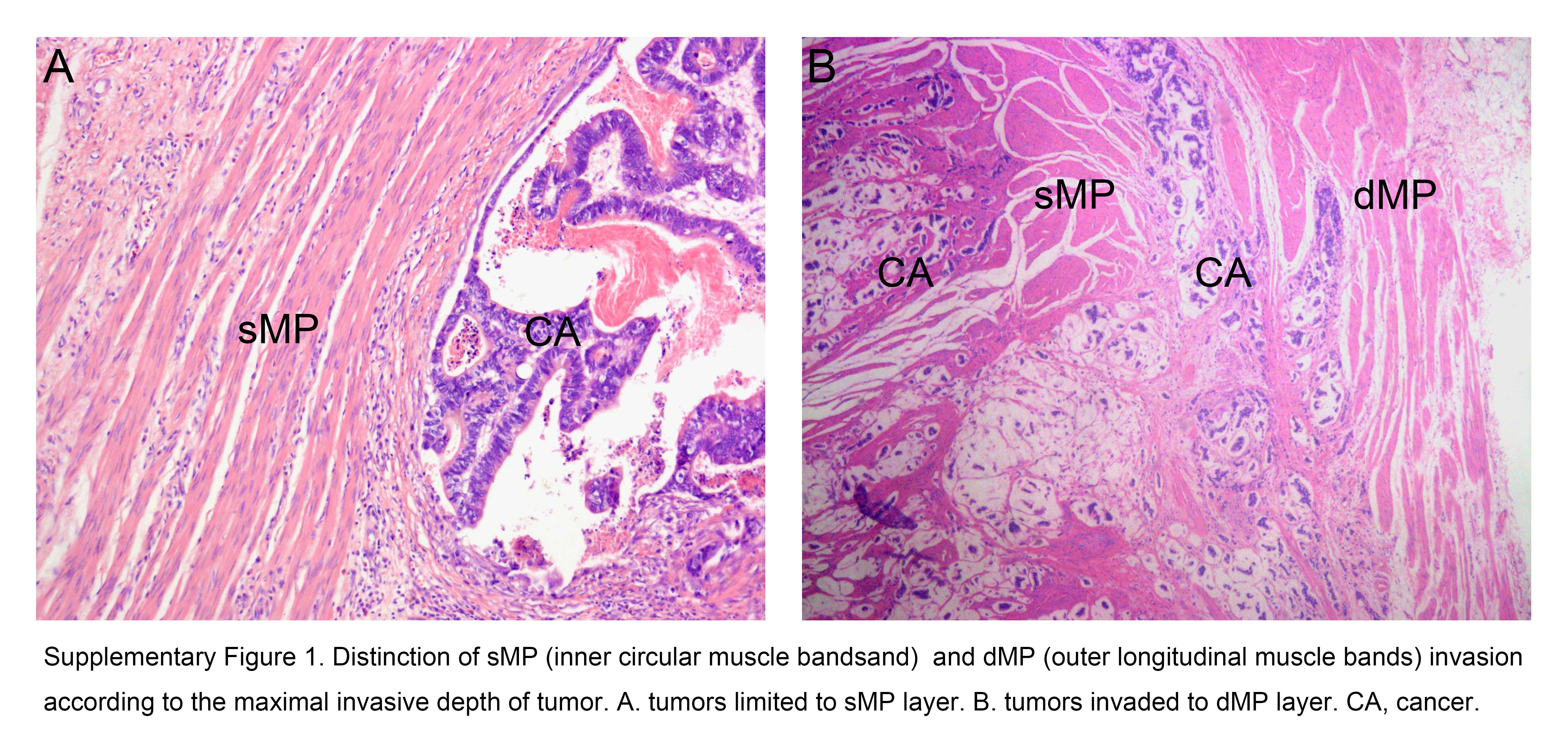

Supplement: Supplementary file 1 — Additional file 1. Fig. S1: Distinction of sMP (inner circular muscle bandsand) and dMP (outer longitudinal muscle bands) invasion according to the maximal invasive depth of tumor. A. tumors limited to sMP layer. B. tumors invaded to dMP layer. CA, cancer. [file 12876_2021_2090_MOESM1_ESM.jpg]

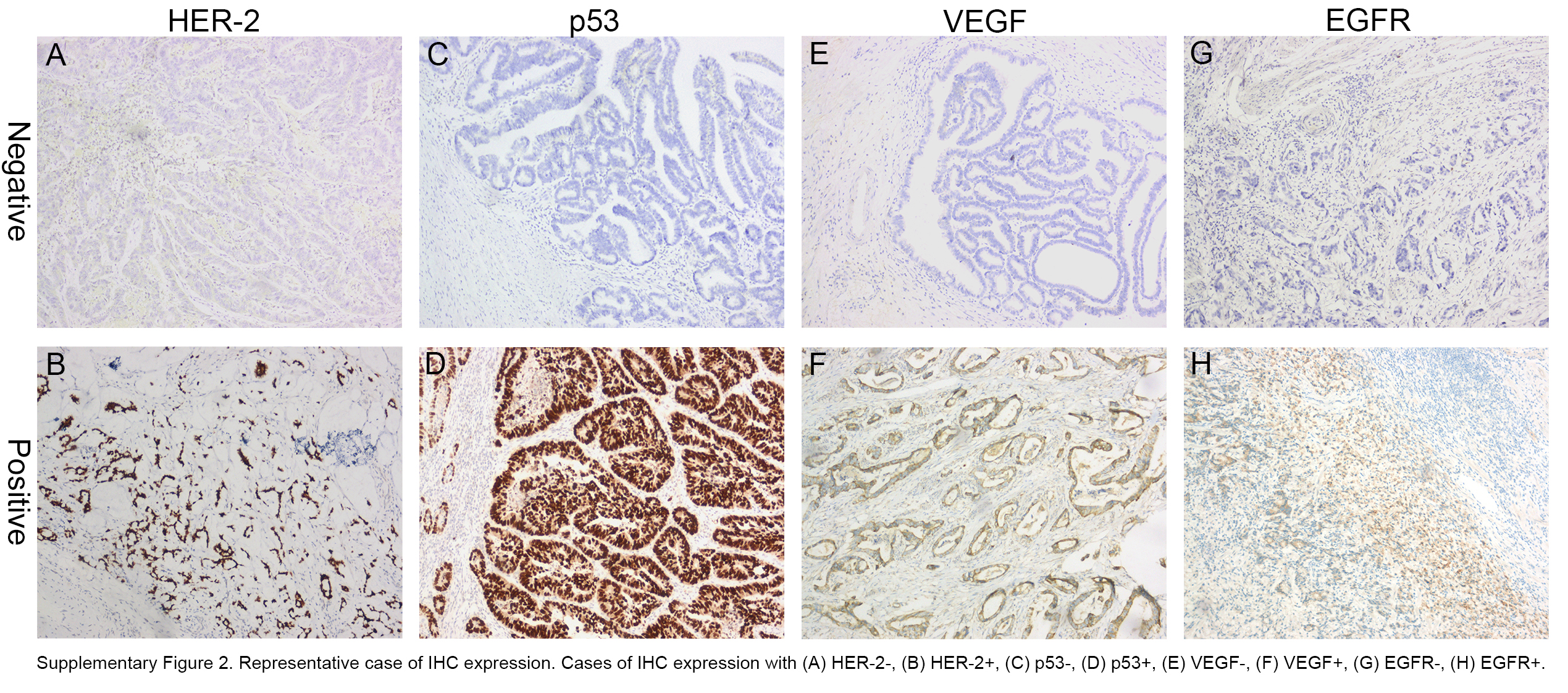

Supplement: Supplementary file 2 — Additional file 2. Fig. S2: Representative case of IHC expression. Cases of IHC expression with (A) Her-2-, (B) Her-2+, (C) p53-, (D) p53+, (E) VEGF-, (F) VEGF+, (G) EGFR-, (H) EGFR+. [file 12876_2021_2090_MOESM2_ESM.jpg]

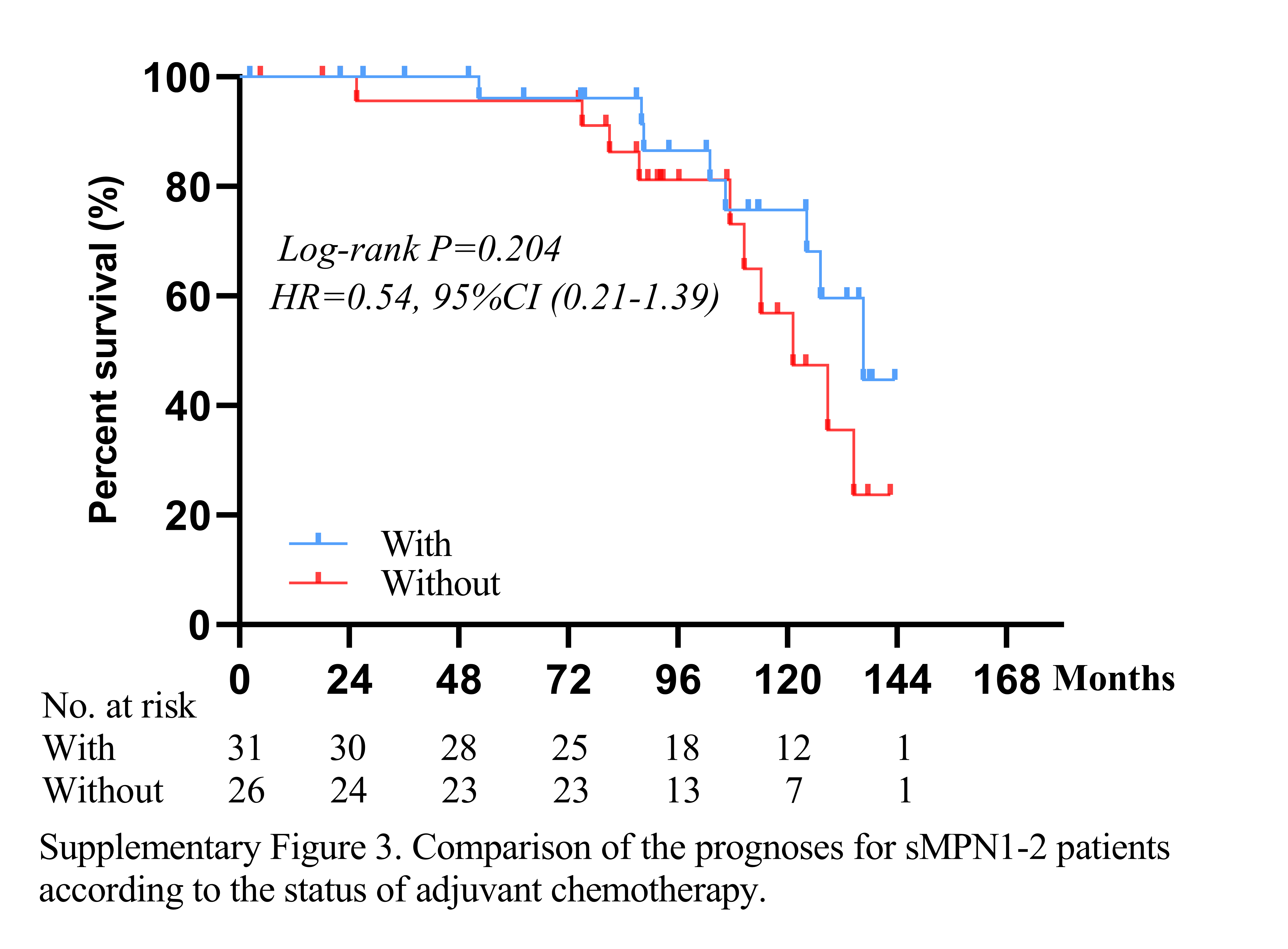

Supplement: Supplementary file 3 — Additional file 3. Fig. S3: Comparison of the prognoses for sMPN1-2 patients according to the status of adjuvant chemotherapy. [file 12876_2021_2090_MOESM3_ESM.tif]

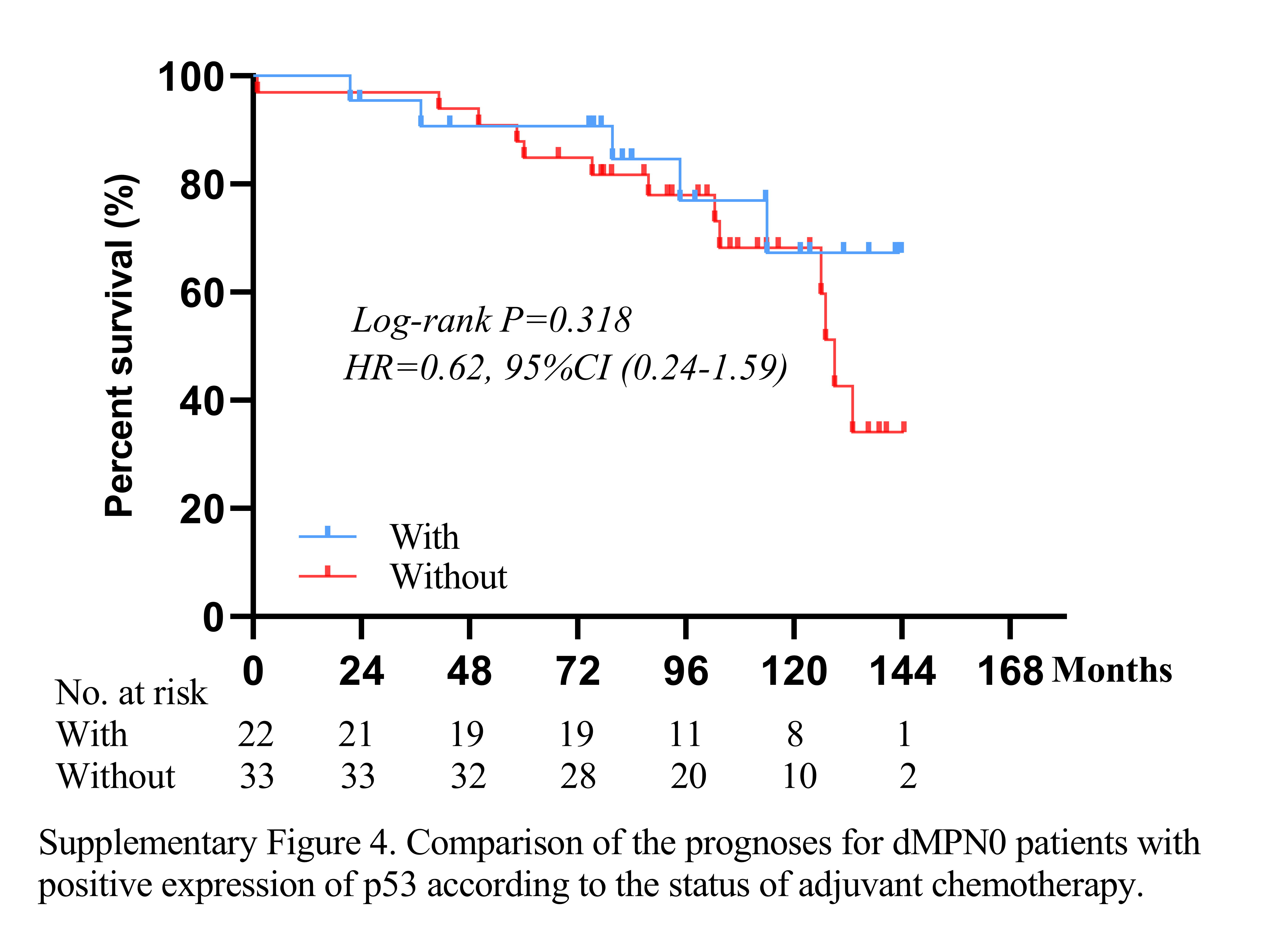

Supplement: Supplementary file 4 — Additional file 4. Fig. S4: Comparison of the prognoses for dMPN0 patients with positive expression of p53 according to the status of adjuvant chemotherapy. [file 12876_2021_2090_MOESM4_ESM.tif]
